# Corrective measures to reduce the accident rate in a company cleaning services sector

**DOI:** 10.47626/1679-4435-2024-1266

**Published:** 2025-01-31

**Authors:** Wilder Alfonso Hernández-Duarte, Paula Andrea Nonzoque-Vertel, Genny Patricia Sánchez-Sierra, Silvana Hiscela Calderón-Aucu, Iván Darío Gaitán-Díaz

**Affiliations:** Corporación Universitaria Minuto de Dios, Bogotá, Colombia

**Keywords:** accidente de trabajo, empresas de aseo, gestión de la seguridad, gestión del riesgo, accidents, occupational, urban cleaning enterprises, safety management, risk management

## Abstract

**Introduction:**

The accident rates presented by organizations are a problem in different economic sectors which should be managed under the principles of continuous improvement typical of occupational safety and health management systems.

**Objectives:**

To define actions on the source, the environment, and the worker aimed at reducing accident rates in a company of the cleaning service sector.

**Methods:**

The accident rate was analyzed through the consolidation of a database provided by the company corresponding to the city of Bogota, Colombia during the 1^st^ semester of 2023. For the analysis of the basic causes of the occupational accidents reported, personal and work-related factors were identified, consolidating the data through Pareto diagrams. Subsequently, actions were taken on the source, the environment, and the worker.

**Experience report:**

In personal factors, 80% of the causes were due to insufficient practices and lack of attention. In the work-related factors, 45.2% of the accidents corresponded to unspecified work factors, i.e., there was not enough information to identify them. This involved analyzing the company’s induction and training practices. By the end of 2023, only four accidents were reported.

**Conclusions:**

From the analysis of the basic causes of the accidents reported, the need to modify the methodology of induction and prevention training sessions was identified, taking into account workers’ sociodemographic characteristics.

## Abstract (es)

**Introducción:**

Los índices de accidentalidad que presentan las organizaciones son una problemática en diferentes sectores económicos que se debe manejar bajo los principios de mejora continua propios de los sistemas de gestión de la seguridad y salud en el trabajo.

**Objetivos:**

Definir acciones sobre la fuente, medio y trabajador para la disminución de los índices de accidentalidad en una empresa del sector de servicios de limpieza.

**Métodos:**

Se analizó la accidentalidad presentada mediante el consolidado de una base de datos suministrada por la entidad correspondiente a la ciudad de Bogotá, Colombia durante el 1^er^ semestre del año 2023. Para el análisis de las causas básicas de los accidentes de trabajo presentados, se realizó la identificación de los factores personales y factores de trabajo y se consolidó los datos a través de diagramas de Pareto. Posteriormente, se concretaron acciones sobre la fuente, el medio y el trabajador.

**Relato de experiencia:**

El 80% de las causas relacionadas con factores personales fue por prácticas insuficientes y falta de atención. En cuanto a los factores del trabajo, 45,2% de los accidentes correspondieron a factores de trabajo no especificados, es decir, no se contaba con información suficiente para su identificación. Lo anterior implicó el análisis de las prácticas de inducción y capacitación de la empresa. A cierre de 2023 se reportaron solo cuatro accidentes.

**Conclusiones:**

A partir del análisis de las causas básicas de los accidentes presentados, se identificó la necesidad de modificar la metodología de las capacitaciones de inducción y prevención, teniendo en cuenta las características sociodemográficas de los colaboradores.

## INTRODUCTION

The International Labor Organization defines occupational accidents (OAs) as unplanned occurrences which result in injuries, fatalities, loss of production, or damage to property and assests.^1^ In Colombia, OAs are considered sudden events that occur due to or on the occasion of work and that produce an organic injury, a function or psychiatric disturbance, a disability, or death in the worker. OAs are also those that occur during the execution of orders from the employer or contractor during the execution of a labor under their authority, even outside the place and hours of work.^2^

According to reports issued by the Colombian Safety Council (Consejo Colombiano de Seguridad, CCS), 542,983 OAs occurred in Colombia during 2022, with a rate of 4.65 OAs per 100 workers, which accounts for 1,488 events per day. The domestic sector presents a rate of 1.88 OAs per 100 workers. For the first quarter of 2023, it is important to highlight the increase in the cases reported, totaling 136,299 accidents, with a rate of 1.17 OAs per 100 workers in general, which accounts for 1514 events per day; according to the aforementioned figures, the domestic sector shows a rate of 0.90 OAs per 100 workers.^3^ Similarly, statistics about accident rates reported for the 2022-2023 period in Bogotá reveal an increase in the number of OAs, with 154,246 cases for 2022 and 34,647 for the 1^st^ quarter of 2023.^3^

Since work-related accidents are one of the main current concerns of companies, due to their negative consequences and high frequency, some guidelines have been established with the purpose of improving the occupational health of the working population. However, despite efforts towards the implementation of new and improved regulations, accident rates in some companies do not show a significant reduction; therefore, some countries have conducted studies to determine the factors that could cause these accidents, as well as those presented in the workplace.^3^

Within this context, Gómez et al.^4^ conducted a cross-sectional study that aimed to estimate the relationship between weekly working hours and occupational injuries, considering a sample of 1,049 Equatorian workers from all economic sectors affiliated with the social security system in 2017. The results of this study show that long working hours are associated with occupational injuries especially in men, among those who worked 44 or more working hours lead to a three-fold increase in the risk of suffering injuries compared to those who work 43 or less weekly hours (equal to or less than 8.6 hours per day).

Furthermore, a study was conducted in 2022 to investigate whether the implementation of a program including the concept total worker health (TWH) reduced absenteeism and work accidents. As a result, there was a 39% decrease in absenteeism in 2018 (absenteeism rate of 1.1) compared with 2017 (absenteeism rate of 1.8); for OAs a reduction of 50% and 81% was observed in days of absence due to OAs after the implementation of the TWH program.^5^

Similarly, in 2014 Zapata & Grisales^6^ conducted a review on the importance of training in the prevention of workplace accidents and found that the main element for creating, practicing, and strengthening the culture of occupational safety and health is workplace-based training, insofar as employees who develop these skills will be able to make decisions towards safe acts, thus reflecting self-management of their safety and health.

Considering the aforementioned general points, it is important to emphasize that the organization with which this project was developed belongs to a business group that operates nationally and whose main economic activity is general interior cleaning of buildings and industrial facilities; however, the present study was conducted based on the reports from the city of Bogotá, Colombia.

According to the statistical records shared by the company, there was an increase in accident rates from January to December 2022, with a mean frequency of 0.58 accidents per 100 workers and a mean severity rate of 2.02 days per 100 workers. For the 1^st^ semester of 2023, the mean frequency rate was 1.82 accidents per 100 workers, and the mean severity rate was 3.11 days per 100 workers. Moreover, it is important to highlight that the greatest number of accidents experienced by workers were caused by blows by an object, entrapment, bruises, falls from one’s own height, among others with a lower incidence (according to the statistical records provided by the entity participating in the research project).

Faced with this situation, the company, in order to comply with the current legal provisions in Colombia, have conducted the respective process with the purpose of reducing accident rates during the period from January 2022 to June 2023, implementing strategies such as: 1) lectures and training sessions aimed at prevention and self-care related to biomechanical, biological, chemical and psychosocial hazards and security conditions; 2) implementation of the campaign named Segurito, which consists of reporting, through a QR code, unsafe acts and conditions of each of the aforementioned risk factors; 3) dissemination of the lessons learned to all employees, based on the accidents reported, through posters and publications in the internal channels of the company.

After the implementation of these actions, frequency and severity rates continued to exhibit an upward trend, which evidences that the implemented measures are not generating the expected results.

In view of the foregoing, this project presents the corrective actions that have contributed to reduce accident rates in the company, analyzing the basic causes of accidents reported during the 1^st^ semester of 2023. Subsequently, actions on the source, environment, and worker were proposed, aimed at intervening in the occurrence of these work-related events that happened in the organization, in the context of continuous improvement. Furthermore, the partial results achieved are described.

## METHODS

The present report adopted an investigative approach in which it was considered relevant to employ a quantitative focus and a descriptive scope by analyzing accident rates using reliable statistical data measured through variables established deductively.^7^ Based on the results, corrective actions were defined and the partial effects of its implementation in the organization were described.

The research proposal was presented to the company for its approval and authorization to provide the database for analysis. This implied a detailed examination of the investigation forms of 42 accidents reported in the company’s database for the city of Bogotá during the first semester of 2023.

The database created in Excel described variables such as days of disability, accident severity, part of the body affected, and type of injury, but did not identify the basic causes of the accidents. For this reason, these causes were classified by the researchers, who divided them into personal and work-related factors, considering the description of the accident and the recommendations proposed by the company. Subsequently, the respective classification was performed according to the criteria established in the Norma Técnica Colombiana (NTC) 3701 of 1995,^8^ which provides guidelines for classification, recording, and statistics of OAs and occupational diseases.

After the classification, data were processed by characterizing accident rates and analyzing the basic causes using the Pareto chart, which is based on the 80/20 principle that establishes a correspondence relationship between the groups, in which 80% of the consequences derive from 20% of the causes.^9,10^ This comparison made it possible to identify and focus on the few vital factors, distinguishing them from the many useful factors.

Based on the results obtained through the analysis of the Pareto chart, the basic causes of the accidents were determined, and the most recurrent variables were established so as to propose corrective actions on the source, environment, and worker for their subsequent application.

For the development of this investigation, the project and its purpose were informed to the company, the corresponding letter of authorization for the study was obtained. Subsequently, the project was validated by the professor who led the hotbed of research named “Management of occupational and health conditions,” the context in which the present project was developed. It is important to highlight that the company will not be affected in any aspect, since complete confidentiality of the information provided.

This report aimed to identify possible failures in the strategies implemented in the aforementioned company and to mitigate or reduce OAs presented in its work environment as well as to reduce economic losses for the company and absenteeism caused by the accidents and thus ensure that employees have safer workplaces that improve well-being during the performance of their activities.

## EXPERIENCE REPORT

From the database provided by the organization, which included all OAs that occurred in the company nationwide, the 42 cases reported in the city of Bogotá during the 1^st^ semester of 2023 were selected.

Table 1 shows the relationship between the part of the body affected and percentage of days of disability. The analysis revealed that the part of the body most affected by OAs were lower limbs (lower limbs, foot, and leg), accounting for 26.2% of the accidents and generating 63 days of disability. This type of accident was often caused by blows with objects and falls of persons or objects, which were due to factors such as lack of attention and insufficient practice. The second most affected part of the body were upper limbs (arms, fingers, and hands), accounting for 21.5% of the OAs and generating 26 days of disability. This type of accident was often caused by blows with objects, falls from one’s own height, and entrapments, and were due to factors such as lack of use personal protective equipment. To explain these results, it bears considering that these parts of the body are the most exposed ones, according to the type of work performed. Furthermore, multiple injuries (i.e., to several parts of the body) accounted for 19% of accidents, generated 29 days of disability, and were often caused by falls from one’s own height, overexertion, false movements, and blows with objects; additionally, these injuries were due to factors such as lack of attention and insufficient practice.

**Table 1:** Relationship between part of the body affected and days of disability

Part of the body affected	n	%	Days of disability by affected part
Abdomen	1	2.4	2
Arm	2	4.8	4
Head	4	9.5	8
Face	3	7.1	7
Fingers	2	4.8	0
General injuries and others	1	2.4	4
Multiple injuries	8	19.0	29
Hand	5	11.9	22
Lower limbs	4	9.5	15
Foot	5	11.9	45
Leg	2	4.8	3
Chest	1	2.4	2
Trunk (back, spine, spinal cord, pelvis)	4	9.5	8
Total	42	100.0	149

The aforementioned injuries increased absenteeism rates and reduced productivity in the company, due to disability-related absences from work; moreover, there was an increase in workload for the other employees.

Table 2 presents the relationship between accident severity and type of injury. The results of this table showed that most OAs (95.2%) reported in the company during the study period were mild (i.e., the employee did not suffer a severe life-threatening injury) and were caused by falls from one’s own height due to slips, badly placed work items, and wet floor. Injuries resulting from blow, contusion, or crushing accounted for 71.4% of the accidents, followed by wounds, with 9.5%, and superficial trauma, with 7.1%. However, the relevance of the other accidents and types of injuries should not be ignored.

**Table 2:** Relationship between severity of accident and type of injury

Severity of the accident	n	%
Mild	40	95.2
Severe	1	2.4
Serious	1	2.4
Total	42	100.0
Type of injury		
Blow, contusion, or crushing	30	71.4
Wound	4	9.5
Superficial trauma	3	7.1
Sprain, strain, muscle tear, hernia, or muscle laceration	2	4.8
Others	2	4.8
Poisoning, acute poisoning, or allergy	1	2.4
Total	42	100.0

To analyze the basic causes of the OAs reported, personal and work-related factors were identified, and data were consolidated in a Pareto chart.

**Figure 1:**
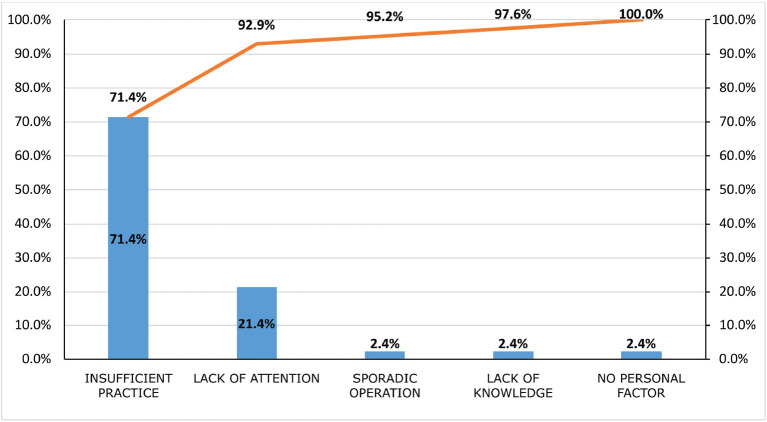
Pareto diagram. Percent distribution of basic causes of accidents by personal factors.

With data analyzed and consolidated through the Pareto chart presented in Figure 1, it was possible to identify the most common basic causes in terms of personal factors. Workers’ insufficient practice is the most representative cause for the occurrence of OA, being the cause of 71.4% of accidents, followed by lack of attention, with 21.4%. This identification is essential to establish relevant corrective measures that allow for reducing accident rates.

It is important to consider that the company’s induction and training plan addresses topics such as: awareness on the use of chemical substances; mechanical, biological, physical, location, and psychosocial risk factors; postural hygiene; free time management; assertive communication; teamwork; and stress management. Of note, the majority of were women aged from 30 to 50 years old with primary school education and, for disseminating its training sessions, the company performed activities such as lectures with highly trained professionals according to the type of risk, sharing of the lessons learned through company’s communication channels, awareness campaigns about unsafe actions and conditions and about the importance of reporting them through QR code implemented in the campaign named “Segurito”. Taking the aforementioned information into account, it is known that, to date, the organization has not implemented a training program that considered variables such as employees’ sociodemographic profile (age, educational level, sex, among others).

**Figure 2:**
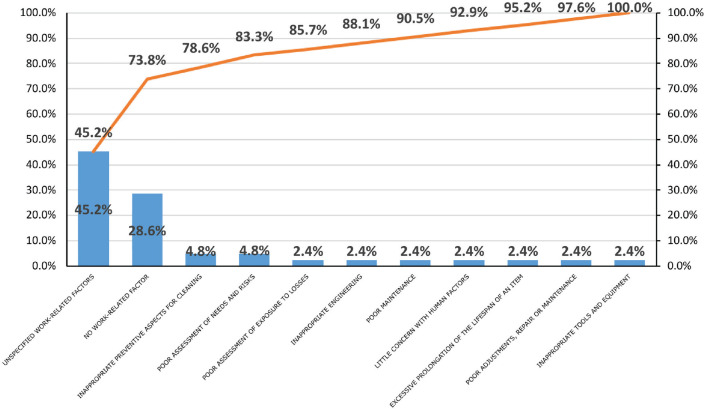
Pareto chart. Percent distribution of basic causes of accidents by work-related factors.

The analysis consolidated in the Pareto chart on the main causes linked to work-related aspects (Figure 2) revealed that 45.2% of the accidents were caused by unspecified work-related factors. Considering the table of OA codes, there was not enough information to classify these accidents into the groups defined in NTC 3701. No work-related factors were observed in 28.6% of the accidents; therefore, it is recommended that the company perform a more detailed analysis and classification of the basic causes of OAs so as to determine whether they should be included in personal factors. Finally, 9.6% of accidents were related to inappropriate preventive aspects for cleaning and poor assessment of needs and risks, which are unsafe conditions related to the work environment and to unsafe acts that depend completely on the person.

Next we present the corrective measures identified and the partial results of their implementation.

Table 3 shows the consolidation and comparison of the basic causes identified, the actions that have been applied, and the corrective actions proposed based on the analysis of the events. Based on the analysis of accident rates in the company, it was determined that corrective actions on source, environment, and worker should be implemented. The results obtained showed that the greater number of accidents reported in the company is due to the workers’ insufficient practice and lack of attention. Since the organization conducts training sessions and inductions, they are recommended to be performed assertively, with a non-technical language that could be clearly understood by the company’s staff, considering sociodemographic characteristics such as educational level and age. Furthermore, it is recommended that the implemented resources be adequate and didactic, achieve to capture the attention, and produce significant impacts that contribute to employees’ proper and effective learning.

**Table 3:** Corrective actions

Cause	NTC 3701 classification	Current actions	Corrective actions		
			Source	Environment	Worker
Use of methods or procedures hazardous per se. Inappropriate placement of personnel (without considering physical limitations, skills, etc.). Lack of knowledge.	Insufficient practice	Induction and training Processes. Training in biomechanical risk factors. Training in active pauses and workplace physical activity.	Execution of processes of induction and training in smaller groups (maximum 100 people). Field visits to verify the application of safety practices. Dissemination of corrections and lessons learned in all areas in the company with teaching aids in order to ensure that the provided information is understood by the entire personnel, considering their sociodemographic characteristics.		Application of the knowledge disseminated in inductions and training focused on postural hygiene. Appropriation of knowledge on the importance of active pauses and workplace physical activity.
Lack of attention	Overconfidence. Lack of assessment of assignments. Lack of attention.	Investigation of accidents. Implementation of corrective measures and subsequent dissemination of the lesson learned.	Periodical on site verification of training to validate their proper learning.		Awareness of the importance of self-care. Appropriate use of prevention signs and personal protective equipment according to the activity being performed.
Preventive aspects inadequate for cleaning	Risk related to clothes or garment. Defective agents. Operating or working at an unsafe velocity.	Selection of providers whose supplied items complying with standard requirements.	Execution of the required tests and assessments of the shoes provided to workers, in order to guarantee that compliance with requirements for the appropriate execution of workers’ tasks and full compliance with standard requirements.		Attendance at the workplace with the supplies provided by the company. Awareness of the risks of not performing their tasks at the appropriate time and manner.

NTC = Norma Técnica Colombiana.

Due to the large number of personnel that the company manages, training sessions and inductions should be performed in large groups, with hampers proper understanding and reduces the effectiveness of these measures. Therefore, it is recommended to conduct them in smaller groups, in order to ensure that lessons and knowledge are completely understood. There have been discussion on the individuals’ learning styles or preferences according to their characteristics and the relationship between these aspects and application of active teaching methodologies for learning processes that guarantee appropriation and applicability of knowledge.^11^ Hence, knowledge was sought to be acquired through new strategies or methodologies in training programs; furthermore, training groups were proposed that to include no more than 100 people, considering the total number of employees working in the company (approximately 1,500), costs, and time for the execution of the programs.

With regard to environment, periodical inspections at employees’ place of work are suggested in order to guarantee that the conditions under which they perform their activities are optimal and appropriate and that allow for identifying and controlling the risks and hazards to which workers are exposed, in addition to promoting safe actions and reducing the probability of OAs.

Since the highest number of accidents were caused by blows and contusions due to slipping, one recommendation was to review whether the personal protective equipment provided, specifically shoes, comply with safety and quality requirements, so as to guarantee that employees are supplied with items that ensure their safety when performing their duties.

The analysis was concluded with the application of the corrective actions established. By the end of 2023, there was a significant reduction in accident rates, with the report of four events for the month of December. Shoes were verified to guarantee compliance with quality requirements for the execution of cleaning activities and, for training programs, it was decided to assign a group of 15 people per supervisor. These supervisors are trained on a monthly basis for them to replicate training in their small groups.

## CONCLUSIONS AND RECOMMENDATIONS

The present study identified the main causes of the accidents reported during the 1^st^ semester of 2023 in a company whose main economic activity is general interior cleaning of buildings and industrial facilities, with the support of the database provided and of the analysis performed through Pareto charts. It was found that the main cause of the accidents were personal factors such as insufficient practice, accounting for 71.4% of the accidents, and lack of attention, accounting for 21.4%. However, 45.2% of the accidents were caused by unspecified work-related factors, i.e., there was not enough information to identify the root cause of the accident.

Based on the analysis of the basic causes of the accidents reported in the organization during the 1^st^ semester of 2023, strategies on source, environment, and worker were proposed aimed at intervening on the occurrence and repetition of the most frequent work-related accidents in the company. The present study identified the need to modify the methodology of induction and prevention training sessions, reducing the number of employees trained in each session, considering their sociodemographic characteristics (age, sex, educational level, among others), implementing playful active strategies capable of capturing their attention and generating efficient retrospective processes in workers that allow them to identify positives and negative aspects in themselves and to implement improvement actions.

A careful analysis of data on OAs should be performed to identify their basic causes, for their subsequent classification, since a high percentage of OAs were not caused by work-related factors.

To increase the objectivity of the analysis, it is recommended to replicate the study with a larger sample, considering the OAs reported in other venues. Furthermore, the impact should be evaluated in terms of the cost-benefit relationship related to the implementation of new training methodologies and review of personal protective equipment, in order to promote employees’ health and well-being and to mitigate the occurrence of OAs.
